# Chemical toxic exposures and chronic ocular pain

**DOI:** 10.3389/ftox.2023.1188152

**Published:** 2023-08-10

**Authors:** Mateusz Graca, Konstantinos Sarantopoulos, Danielle Bodzin Horn

**Affiliations:** ^1^ Department of Anesthesiology, Rush University Medical Center, Chicago, IL, United States; ^2^ Department of Anesthesiology, Perioperative Medicine and Pain Medicine, University of Miami Miller School of Medicine, Miami, FL, United States

**Keywords:** chemical injury, toxicity, ocular surface, cornea, ocular pain

## Abstract

Chronic ocular pain is a common, debilitating chronic pain condition with significant morbidity and negative impact in patients’ quality of life. Several, diverse types of insults to the ocular surface can lead to acute, and under certain conditions to chronic ocular pain, and these include toxic irritants. Exposure of ocular surface to toxic irritants, in addition to direct tissue injury, carries the capacity to generated intense immune and neuronal responses with hyper-excitability, sensitization and chronic pain. Because, chronic ocular pain subsequent to toxic exposures is relatively unrecognized clinical entity, this brief review highlights pertinent concepts of its epidemiology, pathogenesis/pathophysiology, clinical progression, with recommendations for its clinical management that clinicians may find helpful. Suppression of pain signaling, generating neuronal sensitization, and prevention of chronicity of neuropathic pain is particularly emphasized in this respect.

## Introduction

Chronic ocular pain (COP) is a common and debilitating form of chronic pain, perceived as originating from the ocular surface, and frequently extending to adjacent facial structures; including the eyelids, supra/infra-orbital face, and/or the temporal region (Galor et al., 2015; 2022).

COP manifests as spontaneous pain, frequently accompanied by intensely abnormal sensations (tactile allodynia, allodynia to cold, photo-allodynia/photophobia), as well as orofacial pain, TMJ pain, and headaches. It is associated with significant psycho-social dysfunction and can contribute to an extremely poor quality of life (Galor et al., 2018; 2022; Mehra et al., 2020).

The etiology, pathogenesis, and pathophysiology of chronic ocular pain is variable and includes traumatic, post-surgical, post-LASIK (Theophanous et al., 2015), chemical (Wagoner, 1997; Eslani et al., 2014; Kwok and Chew, 2019; Dua et al., 2020; Hoffman et al., 2020; Soleimani and Naderan, 2020), thermal, or infectious noxious insults (Kaufman, 2008). These insults cause sensitization of the peripheral and central sensory afferent pathways, which generate persistent pain and emotional dysfunction (Guerrero-Moreno et al., 2020; Dieckmann et al., 2022; Galor et al., 2022).

Chronic ocular pain can be persistent and debilitating. It has been suggested that easing the burden of COP requires further exploration of its neurophysiology, diagnostic modalities, preventative and treatment strategies (Mehra et al., 2020; Galor et al., 2022). This requires further elucidating the pathophysiology of COP, identifying relevant pharmacology for its management, as well as identifying targeted therapies onto the ocular surface that may reduce the nociceptive burden and drive to neuronal sensitization.

COP remains extremely difficult to treat partly because of its diverse etiology and pathophysiology, because of insufficient knowledge of its underlying mechanisms, and partly because of the lack of specific treatments. Currently available treatment options include various, mostly systemic, analgesic medications, the use of which is limited by significant untoward side-effects (Goyal and Hamrah, 2016; Galor et al., 2022).

Better understanding of the possible causes of COP, its cause-specific pathogenesis, and treatment options may have a positive impact towards its prevention and therapy. Chronic ocular pain generally remains an underappreciated, underdiagnosed and undertreated disease entity, and many ophthalmologists as well as non-ophthalmologist physicians (such as pain specialists, neurologists, primary care physicians, etc.) are at a loss when they treat patients suffering from eye pain, including chronic pain subsequent to toxic chemical insults to the eye. To their assistance, this review aims to highlight pertinent concepts to ocular pain as a result of toxic chemical irritants to the eye surface. These toxic injuries are common and may have a huge impact on the quality of life of patients, not only from the perspective of loss of vision, but from the perspective of chronic, debilitating pain as well. This brief review will highlight basic concepts pertinent to this condition that should be brought to the attention of non-ophthalmology practitioners.

## Epidemiology

Ocular chemical injuries are more likely to occur in the workplace than at home. Toxic chemical injuries account for the second-most common ocular workplace injuries (10%–25%), behind foreign body ocular injuries (35%–50%). Most injuries are reported in the industrial service injury. Men are at least 2 times more likely to suffer a chemical ocular injury than women. Chemical injuries occurring at home are more common in the pediatric population, particularly involving household cleaning agents, medications, and agricultural chemicals (Bizrah et al., 2019). In all settings, splash injuries account for most chemical ocular accidents (Midelfart et al., 2004; Quesada et al., 2020).

The incidence of developing chronic ocular pain from a toxic chemical ocular injury is largely unknown and likely underreported. There are, however, several case reports of patients suffering from chronic ocular pain after chemical injuries from a variety of agents (Memarzadeh et al., 2004; El-Hofi and Helaly, 2019). In a descriptive study conducted on 149 war veteran patients exposed to sulfur-mustard gas, nearly half (43%) described persistent pain many years after the insulting injury (Ghasemi et al., 2009). Clinical outcomes pertaining to vision loss are dependent on the type and amount of chemical agent involved, the duration of chemical exposure, the depth of chemical penetration through the eye, the involvement of extra/intraocular structures, the time to initiation of treatment, the therapeutic course, and the natural healing response (Dua et al., 2020). It is likely that the incidence and extent of chronic ocular pain formation is dependent on the extent of the ocular injury, the therapeutic course taken, as well as the predisposition of a patient for the development of chronic ocular pain—such as a patient with other chronic overlapping pain conditions. It seems that prompt recognition, and appropriate treatment, including management of the pain, acutely as well as in the context of prevention of its transition to chronicity, may be of great importance in ensuring the best possible outcomes.

## Pathophysiology

The surface of the eye, particularly the cornea, is the most densely innervated tissue in the body with a nerve density up to 600 times that of the skin, and up to 40 times that of the tooth pulp (Rózsa and Beuerman, 1982; Midelfart et al., 2004; Quesada et al., 2020). Peripheral afferent sensory fibers are located only a few microns below the corneal surface, allowing for significantly direct exposure to topically acting chemical irritants. When exposed to toxic chemicals onto the surface of the eye, these sensory fibers become directly activated generating intense nociceptive signaling and acute pain. Activated peripheral nociceptive nerve fibers are further sensitized by the ensuing inflammation of surrounding tissues (Wenk and Honda, 2003). Neuronal activation, and sensitization as a result of the ensuing inflammatory response, and ongoing pain may have a significant impact in subsequent events with the potential of transition to chronicity.

Because the majority of sensory afferent neurons in the cornea are nociceptors (Lele and Weddell, 1959; Beuerman and Tanelian, 1979), the primary manifestation of toxic chemical irritants to the eye surface is induction of pain sensation as a result of nociceptive signals entering the brain via the trigeminal nerves. Yet, other mechanisms ensue, in addition to the perception and expression of pain.

The central axons of the trigeminal afferents innervating the cornea project onto two distinct regions of the trigeminal subnucleus caudalis (Vc), specifically to the rostral caudalis/interpolaris transition region (Vi/Vc) and the more caudally located Vc junction in the upper cervical spinal cord (Vc/C1) (Marfurt, 1981; Panneton and Burton, 1981; Marfurt and Del Toro, 1987). These areas respond to nociceptive afferent signals originating from the eye surface (Meng et al., 1997). It seems that the Vi/Vc projection neurons and the Vc/C1 projection neurons may respond differentially to different noxious stimuli, yet chemical irritants activate specific neuronal populations in both sites (Bereiter et al., 2000). These neuronal populations also have the capacity to recruit endogenous modulatory mechanisms during corneal pain (Hirata et al., 2000). This may result in attenuation of the initial painful phase in some patients, while in other patients (under different circumstances, determined by the interaction of individual genetic background and type of exogenous noxious insult) there is a transition to chronicity, whenever mechanisms such as neuro-inflammation and prolonged central sensitization develop.

Various animal models of pain after toxic exposure to the eye have been proposed as means to study those mechanisms. These models employ instillations of toxic chemical to the cornea of animals, such as alkali, or other irritants, and subsequent evaluation of pathological, pathophysiological and neurobehavioral parameters. These (mostly rat and cat) animal models utilize either objective behavioral methods of measuring presence of ocular pain (ie: increase in blinking) or measure changes in neuron firing via surgical insertion of electrodes at various sites along pain pathways (Meng et al., 1997; Wenk and Honda, 2003). These studies facilitate understanding of the pathophysiology and pharmacology of this condition.

Yet, clinically there is an unmet need for specific (and less invasive) diagnostic tools to detect the insulting neural etiologies in patients suffering from COP (Galor et al., 2022), and this applies to pain after toxic chemical exposures, as well. Neuropathic COP after toxicity to the cornea can be diagnosed using a variety of currently available questionnaires, although most are subjective and invalidated. Further diagnostic information can be measured via slit-lamp examination, corneal staining and evaluation of tear production. Direct measures of nociceptor responses in patients include the use of esthesiometry, proparacaine challenge testing, or *in vivo* confocal microscopy, although these tests are largely limited to evaluating the ocular surface (Goyal and Hamrah, 2016). While functional brain mapping has been shown to elucidate visible changes in patients suffering from chronic pain, it is unlikely that these tests provide significant information that would alter clinical management at this time.

Chemical injuries to the surface of the eye cause direct corneal and eye surface injury, including ulcers, as well as inflammation and subsequent events leading to pain, that under some conditions may become chronic (Wagoner, 1997). Yet, chemical injury to the eye has been also shown to extend beyond the cornea, with damage to the retina and optic nerves (Paschalis et al., 2017).

Chemical toxicity by silver nitrate (Wenk and Honda, 2003) has revealed that chemical toxicity rapidly generates acute inflammation with corneal edema and infiltration of neutrophils. Antidromic conduction of acute nociceptive signaling by chemical toxicity may also result in release of neuroexcitatory neuropeptides (such as vasoactive intestinal polypeptide (VIP)) from peripheral terminals of corneal primary afferents adjacent to those exposed to toxic chemicals, thus extending the inflammation (neurogenic inflammation). In contrast, substance P and calcitonin gene-related peptide do not cause any sensitization of corneal afferent neurons (Belmonte et al., 1991; 1994). This notion may be clinically pertinent considering the lack of therapeutic effects of anti-CGRP agents (used for migraines) in the treatment of corneal pain. Yet VIP may contribute to peripheral afferent sensitization and proper strategies aiming at its blockade may be of value clinically and worthy of future investigations.

This inflammatory response subsequent to chemical toxicity is accompanied by increased sensitivity to stimuli (corneal hyperalgesia) by which application of stimuli produces exaggerated responses. This hyperalgesic response is analogous to the acute inflammatory response with hypersensitivity observed in cutaneous tissues after similar noxious toxicity, including edema, neutrophil aggregation and hyperalgesia. In contrast to cutaneous tissue, however, the cornea is not vascularized, therefore corneal inflammation subsequent to chemical toxicity follows a more delayed and prolonged time course and immune cell (mainly neutrophil) infiltration (Wenk and Honda, 2003). The same model indicates that the initial sensitivity may resolve, as a result of recruitment of endogenous descending inhibitory mechanisms from the CNS, while edema and inflammation may persist. Yet, under conditions of more prolonged and extensive nociceptive input, the inhibitory mechanisms may be overwhelmed and more prolonged peripheral sensitization as well as central sensitization may ensue, leading to chronicity of pain.

This has been supported by another animal model of corneal toxicity, induced by repeated topical installations of 0.2% benzalkonium chloride (BAC) onto the left eye of mice (Launay et al., 2016). This model is well-characterized, and simulates conditions like those observed in humans, with reduced tear production (“dry eye”) and with actual pain in the ocular surface. BAC results in neuro-inflammation, results in neurotoxicity in the trigeminal nerve and in projection neurons, accompanied by enhanced hypertonic saline-evoked eye wiping behavior (consistent with hyperalgesia) (Launay et al., 2016). This model results in observed inflammation, neurotoxicity and increases neuronal (FOS, ATF3) and pro-inflammatory (IL-6) markers in trigeminal ganglion neurons, accompanied by enhanced hypertonic saline-evoked eye wiping behavior (suggestive of hyperalgesia). Several markers, suggestive of neuroexcitatory changes in the primary (trigeminal) and second orders afferents pathways (Vi/Vc, Vc/C1 regions) and in the glia have been identified, all of which play a primary role in the central sensitization that drives and maintains chronic ocular pain (Launay et al., 2016). These phenomena may be considered as clinically relevant, explaining the chronicity and intractability of pain is susceptible patients with established neuronal sensitization and centralization of their chronic pain, including their resistance to peripherally targeted therapies.

Other models that include chemical toxicity by 0.75 N NaOH alkali solution to the cornea have revealed the development of corneal nerve damage with an acute profound loss of corneal nerve density, which is followed by a delayed but aberrant reinnervation. Corneal nerve damage was accompanied by ocular hyperalgesia in that model. Furthermore, several abnormalities have been observed in the reinnervating nerves such as neuroma formation, increased tortuosity, beading, and abnormal branching, indicating the presence of mechanisms that contribute to neuropathic pain, in addition to nociception and inflammation (Cho et al., 2019). Therefore, the notion of significant neuropathic pain mechanisms, subsequent to toxicity, should be highlighted in the context of pathophysiology and clinical treatments after chemical toxicity to the ocular surface.

So, it seems very likely that the initial noxious insult from toxic exposure to the cornea may initiate activation of the corneal nociceptors, their sensitization, and generation of afferent nociceptive signals, and synaptic excitatory input to central nociceptive pathways that mediate the sensation of pain and the establishment of central sensitization. While varying types of chemicals may induce differentiating histopathological injuries and penetrate a variety of ocular layers, it is likely that a common pain pathway is shared among these injuries. Sensory nerve fibers (primarily unmyelinated C-fibers, but also A-δ fibers) originating from the ophthalmic division of the trigeminal nerve are activated directly via chemical activation of free nerve endings, polymodal receptors, via ectopic transmission, or damage to nerve axons inducing pathological pain signal transmission. These signals may originate at any of the terminal ocular branches of the trigeminal nerve, transmit to the trigeminal brainstem complex, then project centrally to the posterior thalamus and a variety of cortical targets including the insular cortex, amygdala, somatosensory and prefrontal cortex (Goyal and Hamrah, 2016). Peripheral sensitization, ensuing from local inflammatory and immunoreactive mechanisms secondary to toxicity, as well as central neuronal sensitization, and neuroinflammation, may further sustain chronicity of pain, as well as parallel events, such as photophobia, and emotional dysfunction (Digre and Brennan, 2012; Galor et al., 2015; 2022). These are overall difficult to treat once established. The mechanism of conversion from acute nociceptive pain to chronic neuropathic ocular pain is not fully elucidated and may be more complex than the formation of chronic neuropathic pain in other peripheral nerves. Peripheral sensitization ensues largely after lasting pathological activation of affected nerves deregulates regular transmission activity. Injured terminal neurons can form microneuromata resulting in ectopic pain transmission (Goyal and Hamrah, 2016). Aberrant neural modulation may also originate at the level of cell bodies in the trigeminal ganglia, similarly to the pathological modulation at the level of the dorsal root ganglia in other peripheral neuropathies. Central sensitization to pain is also a key component of the overall development of chronic pain. Central sensitization begins at the level of the second-order neuron synaptic transmission, via upregulation of largely glutamate receptors. Further central sensitization ensues with activation of neuroinflammatory cascades at the level of the brain, with the activation of astrocytes and microglia. This chronic sensitization eventually leads to maladaptive neuroplasticity which is evident with the development of both experienced patient symptoms and objective findings on neuroimaging (Seifert and Maihofner, 2011; Vehof et al., 2013; Crane et al., 2017). While temporal maladaptive developments have not fully yet been elucidated, there is compelling evidence to suggest that the central sensitization leading to chronic pain development is dependent on plasticity of the mesolimbic-prefrontal circuit, then neocortical reorganization. The functional implications of aversive motivational learning and memory formation associated with neuroplasticity in dopaminergic projections (primarily from the ventral tegmental area), and glutamanergic projections (primarily from the amygdala and hippocampus) are likely the mesolimbic changes adapting to constant pain signalization from the periphery (Mansour et al., 2014). The development of chronic pain has also been associated with central changes co-existing in patients with depression, a development likely directly linked due to the high neuro-inflammatory role of pain-related central sensitization (Krishnadas and Cavanagh, 2012). There are fewer amygdala connections with serotonergic projections in patients with chronic pain as well as with depression (Zheng et al., 2022). Both concomitant states have been associated with global cortical gray matter volume alterations (Ma et al., 2022). Furthermore, the two separate conditions may augment each other, worsening the states of both depression and chronic pain (Surah et al., 2014).

In order to guide treatment efforts, grading the extent of the ocular injury is done via either the Roper-Hall classification and/or the Dua et al. classification systems. The Roper-Hall classification system is based on the extent of corneal injury and limbal conjunctival ischemia. It does not consider direct conjunctival injury. The Dua et al. classification system factors in conjunctival injury (percentage of injury) and limbal injury (graded by surface-area “clock hours”). The Dua et al. classification system does not, however, factor in corneal injury. A significant drawback to both classification systems is that neither system takes into consideration the depth of chemical injury, which is of particular significance when considering the pathological etiology for patients’ presentations of COP (Bizrah et al., 2019). Furthermore, while both classification systems have been trialed independently and comparatively to prognosticate recovery and lead treatment efforts, there has not been an assessment of utilizing these classification systems for the evaluation and management of COP.

### Common toxic chemical ocular injuries

Numerous toxic chemicals in existence can cause ocular damage upon direct exposure to the ocular surface. However, similar pathological reactions are seen within certain groups of chemicals. There are largely 3 different groups of toxic chemicals that induce specific reactions: acids, bases, and “other” chemicals. It is unknown currently whether a certain group of chemicals induces COP with a higher incidence than another group of toxic chemicals.

### Alkali agent injuries

Base, or alkali, chemicals account for more significant ocular injuries (80%) than acidic chemicals. Alkali injuries are particularly significant if the pH of the chemical irritant is > 11.5 (Dua et al., 2020). Alkali chemicals are more prone to causing significant ocular injuries due to their ability to penetrate deeper into the eye than acidic or other chemicals. Alkali chemicals can react with cellular membranes causing saponification and lysis of membranes (via hydroxyl ion release and conversion of cell membrane lipids to salts). This will subsequently cause destruction of the ocular epithelial layer and allowance for penetration directly into the anterior chamber of the eye. Further allowance of penetration into the anterior chamber is mediated by the basic chemical’s ability to hydrolyze the ocular surface interfibrillar glycosaminoglycans and disrupt the intact extracellular matrix. If enough of the corneal surface is damaged, alkali toxins have been known to induce a temporary paradoxical anesthesia of the eye due to significant nerve destruction. Lime-plaster is the most common cause of alkali chemical ocular injury. The toxin within lime is Ca(OH)_2_, a chemical compound commonly used in plastering or cementing. The calcium component produces a soap upon saponification within the epithelium, which hinders further penetration into the eye. Ammonia (NH_3_) is a compound used mostly in manufacturing, in fertilizer, and in the chemical industry. It has the fastest rate of penetration through the ocular surface. Another compound with a fast penetration rate is lye (NaOH), which is a common chemical component in drain cleaning products. Other alkali chemicals commonly encountered include potash (KOH), used in the chemical industry, or Mg(OH)_2_, which is found in some fireworks (Morgan, 1987; Lusk, 1999; Dua et al., 2020).

### Acidic agent injuries

Although very capable of causing catastrophic ocular pathology and subsequent chronic pain, acidic chemicals are generally less damaging when contacting the human eye than alkali chemicals. While most alkali chemicals are capable of deep penetration past the anterior chamber of the eye, acidic chemicals are generally stabilized before penetrating the anterior chamber. Acidic compounds primarily cause denaturation and coagulation of the corneal epithelium. Certain acids, such as Hydrofluoric acid (HF), can penetrate deeper into the anterior chamber of the eye. HF is used for rust removal, brick cleaning, pottery glazing, as a lab reagent, and is sometimes used in the semiconductor industry. Yet, the most common ocular injury from acidic chemicals is due to sulfuric acid (H_2_SO_4_). Sulfuric acid is primarily found in car batteries, fertilizer, or used in the metal industry. Other acidic chemicals that commonly cause ocular injuries include HCl (used in the steel industry or chemical manufacturing) or nitric acid (used in chemical manufacturing) (Morgan, 1987; Lusk, 1999; Dua et al., 2020).

### Other chemical agent injuries

Besides acids and bases, other chemicals can cause devastating ocular injuries and subsequent COP. Some common offenders include hydrocarbon fuels (such as gasoline or kerosene) or hydrocarbon solvents (used as cleaning/dissolving substances or paint thinners). These chemicals generally cause superficial pain upon irritation of the cornea, but they do not generally further penetrate the eye. At low concentrations typically encountered, irritant lacrimators (such as pepper-spray) similarly cause corneal nerve stimulation without further structural injury. Cyanoacrylate adhesives (found in a variety of glue preparations) can cause corneal damage from abrasions due to rapid adherence of the eyelids to the cornea. At household strength, H_2_O_2_ is usually no more than just an irritant to the corneal surface. Similarly, alcohol solutions generally cause limited irritation. Formaldehyde, which is used as a preservative, causes mild irritation initially, but it can potentially penetrate the anterior chamber of the eye if it is not promptly washed out (Morgan, 1987; Lusk, 1999; Dua et al., 2020).

### Evaluation and treatment of patient with neuropathic corneal pain

There are no known definitive ophthalmologic interventions that prevent the development of chronic neuropathic ocular pain after injury from chemical exposure. It is these authors’ belief that the best way to prevent chronic neuropathic ocular pain after injury from chemical exposure is to promptly eliminate the toxic irritant from the ocular surface, to address ocular surface inflammation, and to offer acute pain control. It is important to prevent further ocular pathology, to promote ocular healing, and to manage patient co-morbidities.

After ensuring the patient is hemodynamically stable and without injury to their airway that may require stabilizing interventions, promptly addressing the ocular health of a patient suffering from a chemical splash injury is of paramount importance. Copious irrigation of the eye is, by far, the most important acute intervention to remove the noxious, toxic agent and to prevent complications after toxic chemical exposure to the eye. Irrigation is recommended for at least 30 min. Irrigation with water is an acceptable method, although more balanced solutions (such as lactated Ringer’s) may prevent worsening ocular edema (Dua et al., 2020). Amphoteric chelating agents (ex: EDTA) may be used to neutralize both acid and alkali solutions without causing harmful exothermic reactions. If the injury is secondary to an alkali solution, where deeper penetration is likely, it may be beneficial to utilize hyperosmolar amphoteric solutions (v. iso-osmolar), to achieve deeper irrigating effects and pH control (Soleimani and Naderan. 2020). If the insulting agent is hydrofluoric acid based, irrigating with Hexafluorine ^®^ (an acid (H+) and fluoride (F-) ion absorbent) may yield superior irrigating results. Oil or EDTA may be a preferred irrigating solution for quicklime (calcium oxide) ocular burns (Lorenzana-Blanco et al., 2023). Irrigating efforts should not, however, be delayed in anticipation of obtaining a particular irrigating solution.

While ocular irrigation is imperative to initial treatment efforts of patients with ocular chemical injuries, there must be an emphasis towards ensuring total chemical decontamination of both patients and caretakers. The Occupational Health and Safety Administration (OSHA) outlines an evidence-based decontamination plan that can protect patients and caretakers from further injury after exposure to chemical agents (Department of Labor Logo United States department of Labor, 2023). Prevention of contamination is imperative and involves the utilization of safety equipment (disposable garments, goggles, etc.) and safe work practices (ex: remote sample handling, safe container-opening techniques, etc.). These practices extend to caretakers that respond to patient injuries. Decontamination methods then include physical removal of contaminations (water rinsing, vaporization, scrubbing, steaming), chemical inactivation, disinfection/sterilization, irradiation, and chemical removal (dissolving, surfactant utilization, solidification techniques). Finally, it is important to test for the effectiveness of decontamination, utilizing visual observation, wipe sampling, solution analysis, and permeation testing. Ongoing research continues in improving community awareness towards proper chemical decontamination efforts, the development of novel decontaminants, and addressing novel chemical toxicities. Of note, there is also a priority for future research towards developing methods of decontamination of hair, which may serve as a reservoir for further ocular injury after initial ocular irrigation efforts (Collins et al., 2020; Collins et al., 2021).

After irrigation, an ophthalmologist or emergency medicine provider should instill anesthetic eye-drops and remove any obvious particulate matter on the ocular surface. Adequate acute pain control is imperative for a thorough ocular assessment and continued therapeutic efforts. While not studied directly in the setting of COP, there is evidence to suggest that chronic post-traumatic pain may be avoided by preventing significant central sensitization to pain with adequate pain control in the acute phases of tissue injury (McGreevy et al., 2011). Acute pain control can be achieved with local anesthetic eye-drops and supplemented with oral or IV systemic pain medications. While topical NSAIDS are discouraged in chemical ocular eye injuries, it is unknown whether they are detrimental to ocular healing when administered systemically. Opioid based medications may be significantly beneficial in the treatment of acute ocular pain, which has a primarily nociceptive pathophysiology. However, opioids are unlikely to be beneficial in the treatment of COP, once the pain origins become neuropathic in nature. After achieving adequate analgesia, the ophthalmologist should then perform a thorough ocular assessment, examining the eyelids, cornea, pupil, ocular surface epithelium, stroma, intra-ocular anterior surface segments, iris, lens, intra-ocular pressure, and fundus. The extent of the injury is classified by either the Dua classification or Roper-Hall classification system (Dua et al., 2020). The eye should be swabbed for cultures to rule out infections. Any necrotic tissue is then debrided. Broad spectrum topical antibiotics (ex: fluoroquinolone) is often instilled to prevent infections in the presence of epithelial defects (Dua et al., 2020).

At this juncture the ophthalmologist’s therapies will be targeted to reduce further ocular inflammation, prevent further corneal melting, and management of potential increases in intra-ocular pressure (IOP). Steroid eye drops (dexamethasone or prednisolone) can be utilized to reduce inflammation and minimize proteolytic enzyme release within the cornea. It is of interesting note that topical NSAIDs are discouraged to manage inflammation, as they may contribute to further corneal melting. Sodium citrate eye drops, and oral tetracycline have also been shown to suppress the release of damaging proteolytic enzymes. IOP is managed with eye drops or laser therapy (iridotomy, trabeculoplasty, or cyclophotocoagulation). In certain instances, high IOP requires surgical management (MIGS, trabeculectomy, or shunt insertion) (Bizrah et al., 2019; Dua et al., 2020).

After initial ocular stabilizing interventions and inflammation control, the ophthalmologist’s therapies are concentrated on the promotion of corneal re-epithelialization. Minimal ocular corneal irritation may be treated with solely lubricating eye-drops, however more extensive corneal damage would benefit from additional interventions. Current strategies for the promotion of corneal re-epithelialization include topical autologous peripheral blood-serum therapy, topical umbilical cord serum therapy, topical platelet rich plasma (PRP) therapy, or amniotic membrane transplantation. If there is a deficiency in limbal stem cells from extensive chemical injury, a limbal stem-cell transplantation can be considered to promote re-epithelialization (conjunctival-limbal autograft, *ex-vivo* cultivated limbal epithelial transplantation, or a simple limbal epithelial transplantation). Finally, a variety of corneal transplants (keratoplasty or keratoprosthesis) may be considered (Bizrah et al., 2019; Dua et al., 2020).

As it pertains to the evaluation and management of a patient presenting to a pain physician for concerns of chronic ocular pain after chemical injury, a thorough review of the patient’s relevant medical history and ophthalmologic interventions is of paramount importance. Before considering COP, an ophthalmologist should rule out other underlying ocular pathologies that may be contributing to the patient’s pain. Other neuropathic conditions, such as post-herpetic neuralgia or trigeminal neuralgia, should also be considered (Moshirfar et al., 2022). The patient should have a recent slit-lamp examination with the use of dyes (such as fluoresceine) to evaluate the general health of the corneal surface. A simple Schirmer’s test should be recorded to test for the state of the lacrimal system and assess and quantify actual dryness. A proparacaine challenge is an in-office test that can be utilized to differentiate between peripheral and central neuropathic pain. If the pain is peripheral in nature—or originating at the level of corneal nerves—it should be effectively reduced by topical anesthesia with a topical local anesthetic (proparacaine), while central neuropathic pain would largely remain unchanged. Some centers are capable of esthesiometry, an exam which detects the mechanical nociceptor response and quantifies pain fiber function. *In-vivo* confocal microscopy can also be ordered to image the cornea at a cellular level and study regenerative changes of corneal nerves (ex: nerve sprouting, nerve fiber density, or neuroma formation) (Goyal and Hamrah, 2016; Dieckmann et al., 2017; Moshirfar et al., 2022).

The management of COP is currently largely underdeveloped. This also applies to COP after toxic chemical injuries to the eye. Therapies are largely concentrated at treating neuropathic-type pain, not-specific to corneal nerves. Specific evidence-based therapies are lacking in current literature. Nevertheless, a multi-step approach at managing the patient’s pain is likely most beneficial. Therapeutic strategies aim to promote ocular surface healing, decrease inflammation, induce appropriate nerve regeneration, manage pain, and manage patient co-morbidities. An interdisciplinary approach is beneficial, as an ophthalmologist may manage ocular healing, a psychiatrist may manage concurrent psychological pathologies that frequent accompanies neuropathic eye pain and may contribute to overall illness and worsened pain control, a pain specialist may manage analgesic efforts, and a general practitioner may manage any other co-morbidities that can contribute to poor healing (Crane et al., 2017). Local therapies such as lid hygiene and warm compresses may be beneficial, as well as therapies geared at increased tear production, like preservative free artificial tears, and/or increased tear retention (i.e., moisture goggles). Anti-inflammatory medications such as topical corticosteroids, oral cyclosporine, tacrolimus, anakinra, topical and/or oral azithromycin or oral doxycycline may also prove helpful. Systemic pharmacotherapy remains one of the most effective methods of pain control for patients with COP. Serotonin-norepinephrine reuptake inhibitors (SNRIs) such as duloxetine (20–60 mg per day), venlafaxine (37.5–150 mg per day), or the newer milnacipran (12.5–50 mg per day) may be helpful and tend to have a better side effect profile when compared to the tricyclic antidepressant (TCA) class medications. However, TCAs can be very effective in treating COP, and nortriptyline (25 mg–100 mg nightly) is an excellent choice as it has less side-effects than amitriptyline (25 mg–100 mg nightly). Another first-line medication would be the anticonvulsant, carbamazepine (400–1,200 mg per day divided into 2-3 doses). A gabapentinoid medication (gabapentin 600 mg–3,600 mg per day divided into 3 doses, or pregabalin 75 mg–200 mg per day divided into 2-3 doses) may be a beneficial complementary medication. Low-dose naltrexone (LDN) is an evolving therapy that has been shown to be effective in treating neuropathic pain by attenuating neuroinflammation and central sensitizing mechanisms leading to chronic pain. If the patient’s pain is refractory to first line pharmacotherapy, LDN may be instituted at doses 1.5 mg–4.5 mg per day. Other medications to consider for refractory COP may include mexiletine (225 mg QD–675 mg per day) or low-potency opioid medications, such as tramadol (50–100 mg once or twice a day) in extreme cases, although opioids should be better avoided (for several reasons including their propensity to induce opioid induced hyperalgesia).

Alternative therapies may be beneficial, particularly if used in conjunction to systemic pharmacotherapy. These therapies may include acupuncture, transcranial magnetic stimulation, or percutaneous electrical nerve stimulation, such as TENS applied peri-orbitally, or scrambler therapy. Nerve regenerative therapies are also described, such as autologous serum eye drops at a concentration of 20%–100%. While other types of regenerative therapies such as platelet rich plasma, nerve growth factor, and umbilical cord serum eye drops may be beneficial, they are not easily accessible at this time in the United States (Goyal and Hamrah, 2016; Dieckmann et al., 2017; Moshirfar et al., 2022). Other interventional therapies that have showed to be potentially effective in the treatment of chronic ocular pain (although not specific to chemical-induced COP) include trigeminal nerve stimulation, intrathecal pump therapy, or nerve blocks. Nerve blocks include peri-ocular nerve blocks of nerves adjacent to the eye, sphenopalatine ganglion blocks or superior cervical ganglion blocks in patients with parasympathecially- or sympathetically-mediated pain, respectively (Galor et al., 2018). Botulinum toxin injections may be useful for patients suffering from COP, particularly with concomitant migraines. More research is needed to determine the efficacy of these interventional therapies for the treatment of COP. Furthermore, there may be associated syndromes with COP that warrant multidisciplinary treatment. Patients suffering from COP may concurrently be suffering from other chronic pain conditions, including depression, fibromyalgia, chronic joint pain, and migraines (with or without ocular auras). It is unknown whether some of these conditions can be caused by COP, but there are underlying shared factors among patients suffering from these chronic pain conditions that may be exacerbated by the development of COP. Furthermore, permanent vision loss is linked with an increased risk for serious injuries, depression, anxiety, delirium, and overall poor quality of life (Haring et al., 2016; Crane et al., 2017; Baksh et al., 2021).

## Conclusion

COP is a debilitating chronic pain condition that is often underdiagnosed and undertreated. It is a common occurrence after significant ocular damage from toxic chemical exposure. Patients with COP following chemical injuries may present to a chronic pain provider, and it is imperative to understand the basic epidemiology, pathophysiology, diagnostic modalities, and treatment options for these patients. Further research is needed to better elucidate the formation of chronic ocular pain after toxic chemical exposure. Additionally, further therapeutic options should be researched to better manage patients with chronic ocular pain.
